# Optimizing Nitrogen Sources in Top Dressing for Wheat: Field Study on Growth, Yield, and Ammonia Volatilization

**DOI:** 10.1155/2024/8882675

**Published:** 2024-09-30

**Authors:** Muhammad Sajid Farooq, Rashid Mahmood, Aaqib Hameed, Sajid Ali, Faisal Nadeem, Tahir Hussain Awan, Ammara Fatima, Zaira Ahmad

**Affiliations:** ^1^ Department of Agronomy Faculty of Agricultural Sciences University of the Punjab, Lahore, Pakistan; ^2^ Department of Soil Science Faculty of Agricultural Sciences University of the Punjab, Lahore, Pakistan; ^3^ Rice Research Institute, Kala Shah Kaku, Sheikhupura, Pakistan; ^4^ Department of Environmental Science Lahore College for Women University, Lahore, Pakistan

## Abstract

In alkaline calcareous soils, ammonia volatilization is the primary nitrogen (N) loss process, resulting in the reduced N use efficiency of crops. This study aimed at assessing the impact of different N sources for top dressing on ammonia volatilization, as well as their effects on wheat growth and yield over two years. In each year, half of the recommended N was applied as a basal dose using diammonium phosphate (DAP) and urea. The remaining half was top-dressed 35 days after sowing with various sources: prilled urea (PU), granular urea (GU), ammonium sulfate (AS), and calcium ammonium nitrate (CAN) in the first year; PU, urea coated with a urease inhibitor from 20 g (VnU-20) and 40 g (VnU-40) leaves of *Vachellia nilotica*, biochar-coated urea (BU), and urease inhibitor paraphenylenediamine-coated urea (PPDU) in the second year. Ammonia volatilization losses were tracked for up to 12 weeks from sowing. Ammonia losses from basal-applied N remained consistent in both years, comprising around 4% of the applied N. In the first year, top-dressed AS resulted in the highest losses, followed by GU, while losses from urea and CAN were statistically similar. In the second year, coated fertilizers showed lower ammonia losses compared to PU, with VnU-40 displaying the least losses, 48% less than PU. Nitrogen concentration in wheat grain and straw exhibited a negative correlation with ammonia losses. The choice of top-dressed N source influenced tillering, biological, straw, and grain yields of wheat. In the first year, CAN provided maximum yield benefits, and in the second year, VnU-20 exhibited 27% more grain yield than PU. These findings suggest that top dressing with coated urea, especially VnU-20, has the potential to reduce ammonia losses, improve crop nitrogen status, and enhance economic yield compared to other nitrogen sources.

## 1. Introduction

Nitrogen (N) is a vital macronutrient crucial for plant growth, often deficient in global soils [1]. Common fertilizers such as diammonium phosphate (DAP), urea, calcium ammonium nitrate (CAN), and ammonium sulfate (AS) address soil nitrogen deficiency. However, fertilizer N use efficiency seldom exceeds 50% due to losses through nitrate leaching, denitrification, and ammonia volatilization. To mitigate these losses, applying the recommended fertilizer dose in multiple splits is suggested [2].

For wheat, the recommended nitrogen (N) dose is typically divided into basal and top-dressing applications [3]. The basal dose, often paired with phosphatic and potassium fertilizers, involves the use of diammonium phosphate (DAP) and mixed NPK fertilizers. Top-dressing alternatives include ammonium sulfate (AS), calcium ammonium nitrate (CAN), and urea, each exhibiting distinct soil chemical behaviors and benefits for crops. AS, characterized by a low salt index and sulfur supply, proves advantageous in calcareous soils where high pH triggers ammonia volatilization. CAN provides balanced N nutrition and enhances calcium supply to crops [4]. While urea is a popular and cost-effective choice, its susceptibility to ammonia losses is notable, particularly in conditions of high soil urease activity, pH, and temperature [5, 6]. Strategies including granule coating with hydrophobic materials and soil urease inhibitors such as N-(n-butyl) thiophosphoric triamide (nBTPT), paraphenylenediamine, or Vachellia nilotica leaf extract are used to mitigate urea-induced losses [6–10].

Numerous studies have investigated the impact of different nitrogen sources when top-dressed onto wheat crops. The outcomes of these studies exhibit significant variations influenced by factors such as the nitrogen source employed, soil type, and the method and timing of application [11–14]. Nevertheless, a significant gap is evident in the existing literature, with a lack of research specifically comparing nitrogen sources for top-dressing wheat, particularly focusing on ammonia volatilization losses. This gap becomes particularly pronounced when considering the increased significance of losses during the top-dressing phase, especially in scenarios where the N fertilizer is surface applied, as opposed to soil incorporation in the basal dose.

To address this research gap, our study was conducted with the primary objective of comparing various nitrogen sources used in top-dressing wheat crops, placing particular emphasis on evaluating ammonia volatilization losses. It is noteworthy that certain nitrogen sources investigated in our study have not been previously compared in the context of top-dressing for wheat cultivation.

## 2. Materials and Methods

To identify the optimal nitrogen (N) fertilizer for top dressing in wheat crops, field experiments were carried out over a two-year period (2021-22 to 2022-23) at the Faculty of Agricultural Sciences, University of the Punjab, Lahore (31°29′34.72″ N and 74°17′51.00″ E). In both years, the experiments were carried out at the same farm, though in distinct and neighboring fields. Uniform agronomic practices were implemented in both years. Before seedbed preparation, composite soil samples were collected from 0 to 15 cm soil depth and analyzed for physical and chemical properties using standard procedures. The analysis results for both years are detailed in Supplementary Table 1.

Following soil sampling, the field underwent a presoaking irrigation of 10-cm depth, and at field capacity, the soil was ploughed twice using a tractor-mounted cultivator, followed by planking. Wheat seeds of Faisalabad-2008 cultivar were line-sown using a Rabi drill. In both years, fertilizers were applied at a rate of 120, 90, and 60 kg·ha^−1^ for N, P_2_O_5_, and K_2_O, respectively. Half of the N dose, along with the full dose of phosphorus and potassium, was applied during seedbed preparation using urea, diammonium phosphate (DAP), and sulfate of potash (SOP) fertilizers. The remaining N was top-dressed according to the treatment plan. In the crop year 2021–22, the first irrigation was applied 78 days after sowing (DAS) on February 16, 2022. The second irrigation was applied at 110 DAS (March 21, 2022). In the year 2022–23, the experimental field received irrigation at 35 DAS (December 28, 2022), 75 DAS (February 7, 2023), and 110 DAS (March 14, 2023). Each irrigation event maintains a uniform depth of 8 cm.

### 2.1. Treatment Plan

At 35 days after sowing, the field was subdivided into plots, each measuring 6.0 m × 6.0 m. The remaining nitrogen (N) dose was top-dressed using prilled urea (PU), granular urea (GU), ammonium sulfate (AS), and calcium ammonium nitrate (CAN) in the first year (2021–22). In the second year (2022–23), the top-dressing involved PU, two types of *V. nilotica* extract-coated urea, biochar-coated urea, and paraphenylenediamine-coated urea (PPDU). The detailed treatment plan is outlined in Table 1. The experiments were conducted according to randomized complete block design (RCBD) where each treatment was replicated thrice.

### 2.2. Preparation of Coated Urea

*Vachellia nilotica* extract-coated urea, denoted as VnU-20 and VnU-40, was prepared following the methodology outlined by Rana et al. [9]. In summary, 20 g and 40 g of dry leaf powder from *V. nilotica* were extracted using acetone. The resulting extract was concentrated to a volume of 2 mL and subsequently coated onto 100 g of urea to create VnU-20 and VnU-40, respectively.

For the production of paraphenylenediamine (PPD)-coated urea, 0.6 g of powdered PPD was dissolved in a minimum volume of acetone. This solution was then coated onto 100 g of urea using the same procedure described by Rana et al. [9] for hydroquinone-coated urea.

The preparation of biochar-coated urea involved the production of rice straw biochar, following the procedure detailed by El-Hassanin et al. [15]. Subsequently, 100-g urea prills were wetted with 2 mL of a 10% solution of Arabic gum. Following this, 6 g of rice straw biochar was added, and the mixture was thoroughly blended by rotating in a mixer drum. Once complete mixing was achieved, the biochar-coated urea was removed from the drum and dried in the shade.

### 2.3. Parameters Studied

#### 2.3.1. Determination of Volatilized Ammonia

During the 12-week period from sowing to the booting stage of the wheat crop, ammonia volatilization was assessed using the enclosure method with polyfoam disc traps, as outlined by Shakeel et al. [16]. Within each plot, two traps were positioned opposite to each other along the circumference of an imaginary circle with a radius of 2 meters, sharing the same center as the square plot.

Each trap consisted of a polyvinyl chloride (PVC) cylinder measuring 17 cm in diameter and 15 cm in height, equipped with two polyfoam disc ammonia absorbers. These discs, measuring 2.5 cm in thickness and 18 cm in diameter, were prepared by washing and wringing twice with distilled water and a solution comprising 14.7MH_3_PO_4_, glycerol, and water mixed in a ratio of 4 : 5 : 91, as detailed by Grant et al. [17]. The soft 18-cm-diameter discs were gently pressed to closely fit within the 17-cm-diameter PVC cylinder. The lower end of the cylinder was inserted into the soil by 1 cm, with the lower disc positioned 5 cm above the soil surface to capture ammonia volatilized from the soil. A second disc, placed 1 cm below the upper edge of the PVC cylinder, acted as a shield against atmospheric ammonia. To prevent rainwater from entering the trap, a plastic plate slightly larger in diameter than the cylinder was affixed 5 cm above the cylinder using brackets.

The polyfoam discs within a trap were replaced every 3 to 4 days. After removal, each disc was individually stored in a polyethylene zipper bag containing 250 mL of distilled water. Subsequently, the discs were rinsed multiple times in the water to extract the absorbed ammonia. The concentration of ammonia in the extract was determined using the sodium salicylate-sodium nitroprusside method, as described by Nelson [18]. The ammonia lost per day was calculated by using the following formula:

(1) $$\begin{array}{c} \text{Ammonia lost}\left(\text{mg }N\;m^{-2}\text{da}y^{-1}\right)=\frac{C\times 250}{1000\times \text{ND}\times 0.025}, \end{array}$$

where *C* is the pad-extracted ammonia N in the aqueous solution (µg mL^−1^). The total volume of the ammonia containing aqueous solution is 250. 1000 = For the conversion ammonia N from µg to mg. ND is the number of days a trap stayed in the field. The surface area of the soil under the trap is 0.018 m^2^.

Ammonia N lost in a week was calculated by aggregating per day values of all the traps used for a single point in a week.

#### 2.3.2. Estimation of Agronomic Parameters

At the physiological maturity of wheat, ten plants were randomly chosen from each plot, and data on the plant height, spike length, the number of spikelets per spike, and the number of grains per spike were collected from all tillers of these selected plants. The means of these measurements were utilized in the subsequent statistical analysis.

For the assessment of productive tillers, a 1-m^2^ area was randomly selected three times within a plot using a quadrant. The productive tillers were counted, and the means were employed in the statistical analysis. At the stage of maturity, all wheat plants in a designated plot (entire plot area 36 m^2^) were manually harvested, bundled, and weighed using a spring balance to record the biological yield in kg·ha^−1^. The bundles were then threshed, and the grains were weighed to determine the grain yield. The straw yield was calculated as the difference between the biological yield and grain yield. Additionally, from each plot, a random sample of 1000 grains was taken and weighed to obtain the 1000-grain weight.

#### 2.3.3. Estimation of Crop Nitrogen Status

A measured quantity of powdered wheat straw and grains underwent digestion with concentrated sulfuric acid and a digestion mixture (K_2_SO_4_: FeSO_4_: CuSO_4_ = 10 : 1 : 0.5). The resulting digests were distilled using a Kjeldahl distillation apparatus to ascertain nitrogen (N) concentration in both wheat grains and straw, following the methodology outlined by Jackson [19]. The nitrogen uptake by grains and straw of wheat was calculated by multiplying their respective nitrogen concentrations with the corresponding yields.

### 2.4. Statistical Analysis

For statistical analysis, data from both years underwent the analysis of variance, and mean comparisons were conducted using Tukey's HSD (*p*=0.05) with Statistix 8.1 software. This approach provided a robust evaluation of the variability within the dataset and enabled meaningful comparisons of the means. Additionally, for ammonia volatilization data, Tukey's HSD (*p*=0.05) was performed to compare fertilizer treatment means during each week for up to 7 weeks after top dressing.

## 3. Results

### 3.1. Ammonia Volatilization Losses from Basal-Applied Fertilizer N

**Figure 1:**
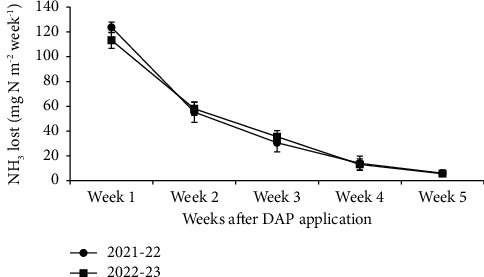
Average ammonia volatilization from the wheat field for five weeks starting from the application of diammonium phosphate (DAP) as the basal dose of nitrogen and phosphorus till the top dressing of nitrogen.

In both years, total ammonia losses from basal-applied fertilizer N were statistically similar, approximately 230 mg·N·m^−2^. These losses accounted for 3.8% of the applied fertilizer N. The highest losses occurred in the first week after sowing and gradually decreased, reaching a negligible value by the fifth week (Figure 1).

### 3.2. Ammonia Volatilization Losses from Top-Dressed N Fertilizers

**Figure 2:**
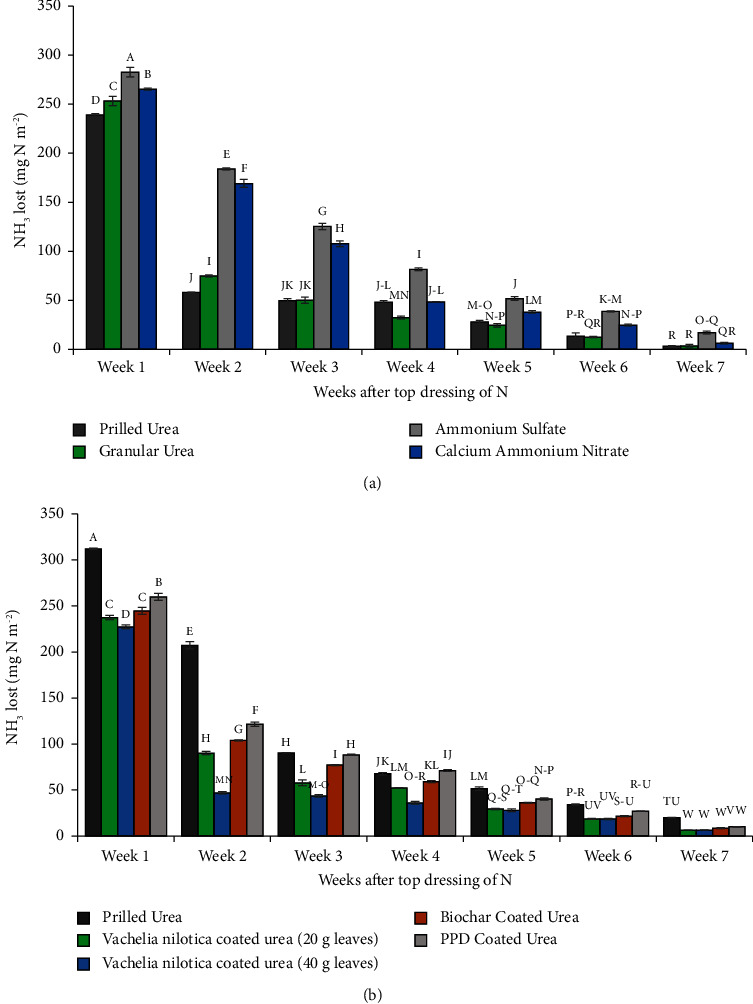
Impact of types of nitrogen fertilizers top dressed after five weeks of wheat sowing on ammonia volatilization from the field soil in Rabi seasons of 2021–22 (a) and 2022–23 (b). Fertilizers are described in Table 1.

**Figure 3:**
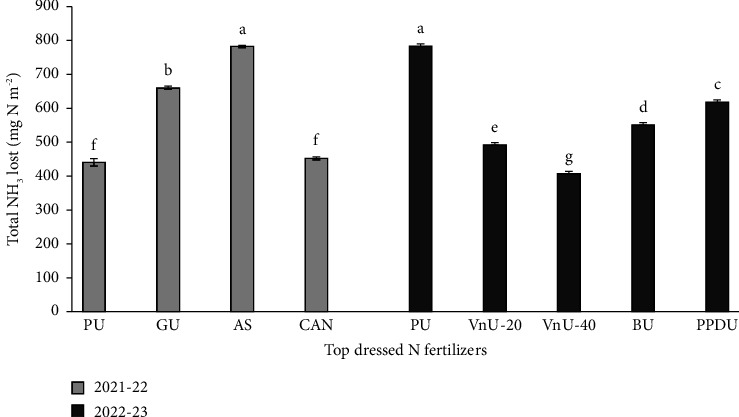
Total ammonia lost after top dressing of various nitrogenous fertilizers to wheat fields in the Rabi seasons of 2021–22 and 2022–23. PU: prilled urea; GU: granular urea; AS: ammonium sulfate; CAN: calcium ammonium nitrate; VnU-20 : 20-g leave's extract of Vachellia nilotica coated to 100-g urea; VnU-40 : 40-g leave's extract of Vachellia nilotica coated to 100-g urea.

In the experimental year 2021–22, the remaining half of the N dose (60 kg·ha^−1^) was supplied through prilled urea (PU), granular urea (GU), ammonium sulfate (AS), and calcium ammonium nitrate (CAN). Ammonia volatilization losses from all top-dressed fertilizers gradually decreased over time. Ammonium sulfate exhibited the highest losses, with 282 mg·N·m^−2^ (4.7% of applied N) in the first week and 17 mg·N·m^−2^ (0.28% of applied N) in the seventh week after top dressing (Figure 2(a)). PU and GU displayed similar trends, with slightly higher losses from GU in the first and second weeks. Although the temporal patterns of ammonia losses from CAN differed from those of prilled urea, the total losses from both fertilizers were statistically similar (Figures 2(a) and 3).

In the following year (2022–23), significantly higher ammonia losses occurred from PU compared to the previous year. During the initial three weeks after top dressing, ammonia losses from PU were significantly higher than those from coated urea fertilizers, except that of PPDU, where the losses were similar to PU only in the third week. Over this period, VnU-40 exhibited the minimum losses, followed by VnU-20, BU, and PPDU (Figure 2(b)). VnU-40 conserved ammonia N in the soil by 27%, 77%, and 51% more than PU in the first, second, and third weeks after top dressing, respectively. However, from the fourth to the seventh week, ammonia losses from all top-dressed fertilizers became statistically similar (Figure 2(b)).

Total ammonia losses from the top-dressed N fertilizers are presented in Figure 3. In 2021–22, AS resulted in the highest total ammonia loss, followed by GU, CAN, and PU. Coated urea fertilizers exhibited lower losses than uncoated PU, with VnU-40 showing the minimum losses. On average, total ammonia losses due to coated urea VnU-20, VnU-40, BU, and PPDU were 37%, 48%, 29%, and 21% less than those of PU, respectively, in 2022–23.

### 3.3. Impact of Choice of N Fertilizer on Growth and Yield Parameters of Wheat

Choice of the N source for top dressing to wheat crops significantly affected the number of productive tillers of wheat in Rabi seasons of 2021–22 and 2022–23. In the first year of experimentation, the maximum number of productive tillers was noted due to CAN, which were 12.8% higher than that of PU. In the case of GU and AS, the number of productive tillers was statistically similar to that of PU. In both the years, parameters, plant height, spike length, number of spikelets per spike, grains per spike, and 1000-grain weight, were not influenced by the choice of N fertilizer top dressed to wheat crops (Table 2).

In the experimental year 2021–22, CAN, GU, and AS demonstrated similar biological yield, straw yield, straw N content, and straw N uptake, all of which were significantly higher than those of PU (Table 2). CAN exhibited the highest grain yield, which was 21.3% higher than that of PU (Supplementary Table 2). GU and AS showed grain yields similar to PU in the same year. The grain N content and grain N uptake were also higher in the case of CAN compared to other top-dressed N fertilizers. On average, the grain N content (%) and grain N uptake for CAN were 29.1% and 58.2% higher than those for PU (Table 2 and Supplementary Table 2).

In the second experimental year (2022–23), key agronomic parameters such as plant height, spike length, number of spikelets per spike, grains per spike, and thousand-grain weight were not significantly influenced by the choice of the N fertilizer used for top dressing in wheat (Table 3). However, the number of productive tillers, biological yield, straw yield, grain yield, grain N content, straw N content, grain N uptake, and straw N uptake were significantly the highest with VnU-20 compared to other fertilizers.

Specifically, in the second experimental year (2022–23), VnU-20 demonstrated significantly higher grain yield, grain N content, and grain N uptake, with increases of 27.3%, 51.3%, and 96.2%, respectively, compared to PU (Table 3 and Supplementary Table 3). In terms of overall crop benefits, VnU-20 was followed by BU and VnU-40. Additionally, compared to PU, several parameters, including the number of productive tillers, biological yield, straw yield, and grain yield, exhibited lower values with PPDU (Table 3).

## 4. Discussion

In the first year of the experiment, the highest N losses were observed from soil treated with ammonium sulfate (AS) as the top-dressed fertilizer. AS contains only the ammonium form of N, which quickly becomes available upon fertilizer application and easily converts to ammonia in the alkaline environment of calcareous soil [20]. While the sulfate ion (SO_4_^−^) in AS has an acidifying effect, potentially reducing volatilization losses by keeping the fertilizer N in the ammonium form and converting less to gaseous ammonia [21, 22], the effectiveness of this acidifying effect depends on the microbial reduction of sulfate to hydrogen sulfide [H_2_S_(g)_] and the formation of hydrosulfuric acid [H_2_S_(aq)_] [23]. This process is slow and might not significantly reduce ammonia loss from AS in our study, where maximum losses occurred within two weeks of fertilizer application. Considering the high ammonia volatilization losses from soil-applied AS, its use as a nitrogen and sulfur source is already discouraged in soils with a pH greater than 7.0 [22].

More ammonia volatilization losses and similar N accumulation and yield were observed with GU compared to PU. These findings diverge from the results reported in several studies showing higher nitrogen use efficiency with deep-placed supergranular urea compared to plain urea, as observed in studies on rice [24] and wheat [25]. The advantage of applying GU over PU seems to be linked to fertilizer application depth [26]. GU significantly increased both the wheat crop N status and yield when applied at 7.5 cm soil depth compared to PU [25]. However, PU performed better when the fertilizer N was applied at 2.5 cm soil depth [27]. Similar trends were observed in pot-cultivated cucumber, where surface-applied PU exhibited greater efficiency in enhancing growth and yield parameters compared to surface-applied supergranular urea [28]. Prilled urea, with its higher surface area, dissolves more rapidly in water than GU, facilitating quicker absorption into the soil. With GU, a greater quantity of N tends to remain on the soil surface, undergoing conversion to ammonia and being more susceptible to easy loss to the atmosphere.

Ammonia volatilization losses from CAN were similar to those from PU, contrary to earlier studies reporting lower losses from CAN compared to urea [29]. The comparable levels of ammonia losses between CAN and PU seem to be associated with the surface application of both fertilizers. When deep-placed, CAN could potentially show lower ammonia losses due to its dual nitrogen forms and hygroscopic nature. However, when applied to the soil surface, its ammonia losses were observed to be as high as those from PU. Some growth parameters of wheat exhibited superior results with CAN compared to urea, likely attributed to the direct supply of nitrate-N in CAN. This enhanced performance aligns with previous studies that have observed the wheat crop's better response to nitrate-N compared to ammonium-N [30].

In comparison with the first year, ammonia losses were higher in the second year of the experiment. This increase in losses can be attributed to the elevated air temperatures during the second year. In the first year, the maximum air temperature was 11°C at the time of top dressing of N fertilizer, rising to 16°C in the second week after top dressing. However, in the second year, the maximum air temperature during top dressing was 14°C, escalating to 18°C in the mid of the first week (Supplementary Figure 1). The rise in temperature contributed to increased ammonia losses [5] by enhancing the rate of diffusion and decreasing ammonia solubility in water.

The application of urea coated with urease inhibitors, specifically paraphenylenediamine (PPDU) and *V. nilotica* extract, proved effective in mitigating ammonia volatilization losses compared to PU. Previous research has established the urease inhibition potential of *V. nilotica* extract, indicating that using an extract from 20 g of *V. nilotica* leaves to coat 100 g of urea (VnU-20) yielded superior results compared to an extract from 10-g leaves [9]. Expanding on this, our study explored the use of a 40-g leaf extract (VnU-40), which demonstrated even greater efficacy in reducing ammonia volatilization losses. However, the use of PPDU and VnU-40 resulted in lesser growth and yield compared to VnU-20, possibly attributed to the toxic effects of the higher doses of these urease inhibitors. The phytotoxicity of various urease inhibitors is already reported in the literature [31].

Ammonia volatilization losses from biochar-coated urea were significantly lower than those from PU. The influence of biochar on ammonia volatilization is contingent upon its pH. Soil-applied biochar with a pH exceeding 7 tends to increase ammonia volatilization losses, while the opposite is observed for biochar with a lower pH [32]. The rice straw biochar used in coating urea had a pH exceeding 7 [15], and therefore, the reduced ammonia volatilization losses from the biochar-coated urea were not due to its pH but instead seem to be attributed to the physical impact of the coating. The biochar was coated to urea by wetting it with a 10% solution of Arabic gum, creating a reasonably robust barrier and transforming the biochar-coated urea into a slow-release fertilizer. The role of slow-release urea fertilizers in mitigating ammonia volatilization losses is well-documented in the literature [33].

Nitrogen fertilizers were top dressed to wheat crops at 35 days after sowing, which was the onset of the tillering stage of the crop. Conserving N at this stage significantly increased the number of tillers more than other growth parameters. A keen observation of the data indicates that an increase in straw and gains yields is attributed to an increase in the number of productive tillers.

## 5. Conclusion

In conclusion, our two-year field experiment revealed that top-dressed calcium ammonium nitrate (CAN) outperformed prilled urea (PU) in reducing ammonia volatilization losses and increasing wheat crop nitrogen status and yield. Additionally, coated urea formulations, particularly those with *Vachellia nilotica* extract (VnU-20 and VnU-40), paraphenylenediamine (PPDU), and biochar, proved effective in minimizing ammonia volatilization, showcasing their potential as viable alternatives to traditional urea fertilizers. However, it is important to note that while PPDU and VnU-40 reduced volatilization, they did not lead to a significant increase in the crop yield. Therefore, based on the observed outcomes, it can be concluded that the most promising options for optimizing nitrogen management and minimizing ammonia losses in wheat cultivation are VnU-20 and biochar-coated urea. These formulations showcase potential as environmentally friendly and efficient alternatives, providing valuable insights for enhancing nitrogen use efficiency while maintaining crop productivity in agricultural practices.

## Figures and Tables

**Table 1:** Description of the fertilizers used to top dress nitrogen in wheat in Rabi seasons of 2021–22 and 2022–23.

Year	Name assigned	Description
2021-22	PU	Prilled urea (Fauji Fertilizers Company Limited)
	GU	Granular urea (Fauji Fertilizer Bin Qasim Limited)
	AS	Ammonium sulfate (FMC)
	CAN	Calcium ammonium nitrate (Fatima Fertilizers)

2022–23	PU	Prilled urea (Fauji Fertilizers Company Limited)
	VnU-20	Extract of 20-g leaves of *Vachellia nilotica* used to coat 100 g of prilled urea
	VnU-40	Extract of 40-g leaves of *Vachellia nilotica* used to coat 100 g of prilled urea
	BU	6-g rice straw biochar used to coat 100 g of prilled urea
	PPDU	0.6 g of paraphenylenediamine (PPD) used to coat 100 g of prilled urea

**Table 2:** Impact of top dressing of nitrogen fertilizers on various growth and yield parameters of wheat in Rabi season of 2021–22.

Parameters	Nitrogen fertilizers used in top dressing				HSD value
	Prilled urea	Granular urea	Ammonium sulfate	Calcium ammonium nitrate	
Number of productive tillers	302.7 ± 2.66b^∗^	305.3 ± 3.48b	316.3 ± 7.26ab	341.7 ± 15.06a	36.18
Plant height (cm)	84.0 ± 3.0	82.7 ± 2.94	87.0 ± 4.01	87.1 ± 2.60	14.33^NS^
Spike length (cm)	10.2 ± 0.27	10.1 ± 0.18	10.8 ± 0.20	10.6 ± 0.24	1.16^NS^
Spikelets per spike	16.7 ± 0.40	16.9 ± 0.43	16.6 ± 0.29	17.3 ± 0.26	1.84^NS^
Grains per spike	40.5 ± 2.84	40.8 ± 3.115	41.4 ± 2.15	41.1 ± 0.34	12.54^NS^
Biological yield (t·ha^−1^)	12.4 ± 0.20b	13.5 ± 0.28a	13.5 ± 0.28a	14.1 ± 0.16a	0.77
Grain yield (t·ha^−1^)	3.3 ± 0.21b	3.4 ± 0.09b	4.0 ± 0.11ab	4.1 ± 0.02a	0.69
Straw yield (t·ha^−1^)	9.0 ± 0.41b	10.0 ± 0.35a	9.45 ± 0.16ab	10.0 ± 0.15a	0.95
1000-grain weight (g)	43.5 ± 0.20	44.4 ± 1.30	45.9 ± 0.38	44.2 ± 0.83	4.22^NS^
Straw N (%)	0.43 ± 0.01b	0.69 ± 0.02a	0.77 ± 0.02a	0.88 ± 0.07a	0.20
Grain N (%)	1.72 ± 0.02c	1.62 ± 0.02c	1.92 ± 0.06b	2.22 ± 0.20a	0.14
N uptake straw (kg·ha^−1^)	39.21 ± 2.15b	69.92 ± 3.73a	73.26 ± 0.61a	88.32 ± 8.27a	19.24
N uptake grain (kg·ha^−1^)	58.38 ± 3.36c	55.42 ± 1.46c	77.89 ± 3.22b	92.37 ± 1.06a	10.06

^∗^Means ± standard error. Means sharing common letters in a row do not differ significantly at *p* > 0.05.

**Table 3:** Impact of top dressing of nitrogen fertilizers on various growth and yield parameters of wheat in Rabi season of 2022–23.

Parameters	Nitrogen fertilizers used in top dressing					HSD
	Prilled urea	*Vachellia nilotica*-coated urea (20-g leaves)	*Vachellia nilotica*-coated urea (40-g leaves)	Biochar-coated urea	PPD-coated urea	
Productive tillers	302.3 ± 4.33b^∗^	363.0 ± 4.04a	294.67 ± 20.78b	303.3 ± 14.72b	235.0 ± 2.64c	54.86
Plant height (cm)	82.9 ± 3.09	82.1 ± 2.14	83.3 ± 3.24	83.3 ± 2.82	82.1 ± 6.71	17.92^NS^
Spike length (cm)	9.90 ± 0.25	10.1 ± 0.28	10.5 ± 0.22	10.1 ± 0.23	10.0 ± 0.56	1.76^NS^
Spikelets per spike	16.4 ± 0.43	16.0 ± 0.26	17.5 ± 0.75	15.4 ± 0.58	15.9 ± 0.27	2.70^NS^
Grains per spike	39.7 ± 2.57	40.0 ± 4.03	40.4 ± 1.41	37.23 ± 3.21	38.3 ± 4.75	16.43^NS^
Biological yield (t·ha^−1^)	12.2 ± 0.20b^∗^	15.5 ± 0.28a	13.0 ± 0.57b	13.5 ± 0.28b	10.0 ± 0.57c	1.84
Grain yield (t·ha^−1^)	3.3 ± 0.19b	4.2 ± 0.06a	3.2 ± 0.01b	4.1 ± 0.16ab	2.6 ± 0.15c	0.62
Straw yield (t·ha^−1^)	8.89 ± 0.40b	11.2 ± 0.35a	9.72 ± 0.58b	9.8 ± 0.19ab	7.3 ± 0.42c	1.42
1000-grain weight (g)	42.2 ± 0.17	43.3 ± 0.85	44.0 ± 0.90	44.1 ± 0.60	43.00 ± 0.48	3.26^NS^
Straw N (%)	0.39 ± 0.01c	0.81 ± 0.02a	0.77 ± 0.02a	0.61 ± 0.01b	0.54 ± 0.01b	0.08
Grain N (%)	1.56 ± 0.02c	2.36 ± 0.02a	2.29 ± 0.02a	2.29 ± 0.02a	1.91 ± 0.02b	0.10
N uptake straw (kg·ha^−1^)	35.09 ± 2.12d	91.09 ± 4.55a	75.16 ± 2.59b	60.03 ± 1.23c	39.82 ± 1.64d	8.83
N uptake grain (kg·ha^−1^)	51.42 ± 2.54c	100.91 ± 1.34a	75.08 ± 0.60b	84.14 ± 4.11b	50.11 ± 3.00c	12.32

^∗^Means ± standard error. Means sharing common letters in a row do not differ significantly at *p* > 0.05. NS: nonsignificant.

## Data Availability

All data used to support the findings of this study are available in supplementary tables and from the corresponding author upon request.

## Supplementary Materials

Supplementary Materials Supplementary Figure 1: Daily mean air temperature and precipitation of the experimental area from wheat sowing to booting stage in 2021–22 (A) and 2022–23 (B). Supplementary Table 1: Physiochemical characteristics of the experimental field soil in 2021-22 and 2022-23. Supplementary Table 2: Percentage increase/decrease with respect to prilled urea in the year 2021–22. Supplementary Table 3: Percentage increase/decrease with respect to prilled urea in the year 2022–23.
