# Supplementary material for: Optimizing Nitrogen Sources in Top Dressing for Wheat: Field Study on Growth, Yield, and Ammonia Volatilization
Source: Scientifica (Cairo). 2024 Sep 30;2024:8882675. doi: 10.1155/2024/8882675 (PMC11458304; doi:10.1155/2024/8882675)
Supplement: Supplementary Materials — Supplementary Figure 1: Daily mean air temperature and precipitation of the experimental area from wheat sowing to booting stage in 2021–22 (A) and 2022–23 (B). Supplementary Table 1: Physiochemical characteristics of the experimental field soil in 2021-22 and 2022-23. Supplementary Table 2: Percentage increase/decrease with respect to prilled urea in the year 2021–22. Supplementary Table 3: Percentage increase/decrease with respect to prilled urea in the year 2022–23. [file 8882675.f1.zip › Supplementary Figure 1.docx]

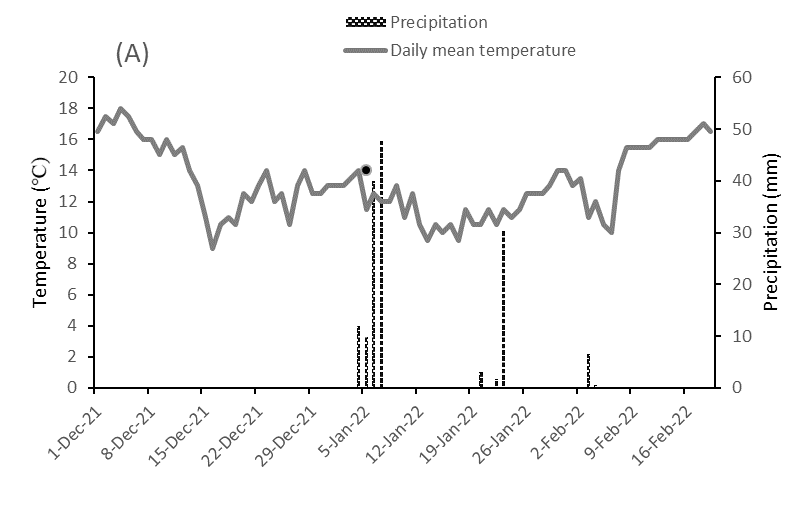


N top-dressing

**Supplementary Figure 1**. Daily mean air temperature, and precipitation of the experimental area from wheat sowing to booting stage in 2021-22 (A) and 2022-23 (B).
