# Supplementary material for: Optimizing Nitrogen Sources in Top Dressing for Wheat: Field Study on Growth, Yield, and Ammonia Volatilization
Source: Scientifica (Cairo). 2024 Sep 30;2024:8882675. doi: 10.1155/2024/8882675 (PMC11458304; doi:10.1155/2024/8882675)
Supplement: Supplementary Materials — Supplementary Figure 1: Daily mean air temperature and precipitation of the experimental area from wheat sowing to booting stage in 2021–22 (A) and 2022–23 (B). Supplementary Table 1: Physiochemical characteristics of the experimental field soil in 2021-22 and 2022-23. Supplementary Table 2: Percentage increase/decrease with respect to prilled urea in the year 2021–22. Supplementary Table 3: Percentage increase/decrease with respect to prilled urea in the year 2022–23. [file 8882675.f1.zip › Supplementary Table 1.docx]

**Supplementary table 1.** Physio-chemical characteristics of the experimental field soil in 2021-22 and 2022-23.

| **Parameters** | **2021-22** | **2022-23** |
| --- | --- | --- |
| Soil sampling depth (cm) | 0-15 | 0-15 |
| Sand (%) | 21.70 | 20.95 |
| Silt (%) | 58.30 | 58.82 |
| Clay (%) | 20.10 | 21.37 |
| Textural class | Silt Loam | Silt Loam |
| Organic matter (%) | 0.62 | 0.67 |
| pH | 7.7 | 7.9 |
| Electrical Conductivity (dS m^-1^) | 2.64 | 2.57 |
| Cation Exchange Capacity ( meq 100g^-1^) | 6.3 | 6.1 |
| Total Nitrogen (%) | 0.03 | 0.04 |
| Available Phosphorus (mg kg^-1^) | 6.46 | 6.82 |
| Extractable Potassium (mg kg^-1^) | 164 | 175 |
