# Supplementary material for: Optimizing Nitrogen Sources in Top Dressing for Wheat: Field Study on Growth, Yield, and Ammonia Volatilization
Source: Scientifica (Cairo). 2024 Sep 30;2024:8882675. doi: 10.1155/2024/8882675 (PMC11458304; doi:10.1155/2024/8882675)
Supplement: Supplementary Materials — Supplementary Figure 1: Daily mean air temperature and precipitation of the experimental area from wheat sowing to booting stage in 2021–22 (A) and 2022–23 (B). Supplementary Table 1: Physiochemical characteristics of the experimental field soil in 2021-22 and 2022-23. Supplementary Table 2: Percentage increase/decrease with respect to prilled urea in the year 2021–22. Supplementary Table 3: Percentage increase/decrease with respect to prilled urea in the year 2022–23. [file 8882675.f1.zip › Supplementary Table 2.docx]

**Supplementary table 2.** Percentage increase/decrease with respect to prilled urea in the year 2021-22.

| **Parameters** | **% Increase/Decrease (2021-22)** | | |
| --- | --- | --- | --- |
|  | **Granular Urea** | **Ammonium Sulfate** | **Calcium Ammonium Nitrate** |
| Total ammonia loss | 50.1 | 77.7 | 2.7 |
| Number of productive tillers | 0.8 | 4.5 | 12.8 |
| Plant height (cm) | -1.6 | 3.5 | 3.7 |
| Spike length (cm) | -1.6 | 5.3 | 3.3 |
| Spikelets per spike | 0.8 | -1.0 | 3.2 |
| Grains per spike | 0.6 | 2.1 | 1.3 |
| Biological yield (t ha^-1^) | 8.9 | 8.9 | 13.7 |
| Grain yield (t ha^-1^) | 0.6 | 18.3 | 21.3 |
| Straw yield (t ha^-1^) | 11.0 | 4.9 | 11.0 |
| 1000 grains weight (g) | 2.0 | 5.4 | 1.5 |
| Straw N (%) | 60.5 | 79.1 | 104.7 |
| Grain N (%) | -5.8 | 11.6 | 29.1 |
| N uptake straw (kg ha^-1^) | 78.3 | 86.8 | 125.2 |
| N uptake grain (kg ha^-1^) | -5.1 | 33.4 | 58.2 |
