# Supplementary material for: Optimizing Nitrogen Sources in Top Dressing for Wheat: Field Study on Growth, Yield, and Ammonia Volatilization
Source: Scientifica (Cairo). 2024 Sep 30;2024:8882675. doi: 10.1155/2024/8882675 (PMC11458304; doi:10.1155/2024/8882675)
Supplement: Supplementary Materials — Supplementary Figure 1: Daily mean air temperature and precipitation of the experimental area from wheat sowing to booting stage in 2021–22 (A) and 2022–23 (B). Supplementary Table 1: Physiochemical characteristics of the experimental field soil in 2021-22 and 2022-23. Supplementary Table 2: Percentage increase/decrease with respect to prilled urea in the year 2021–22. Supplementary Table 3: Percentage increase/decrease with respect to prilled urea in the year 2022–23. [file 8882675.f1.zip › Supplementary Table 3.docx]

**Supplementary table 3.** Percentage increase/decrease with respect to prilled urea in the year 2022-23.

| **Parameters** | **% Increase/Decrease (2022-23)** | | | |
| --- | --- | --- | --- | --- |
|  | ***Vachelia nilotica* coated urea (20 g leaves)** | ***Vachelia nilotica* coated urea (40 g leaves)** | **Biochar Coated Urea** | **PPD Coated Urea** |
| Total ammonia loss | -37.1 | -48.0 | -29.6 | -21.1 |
| Number of productive tillers | 20.1 | -2.5 | 0.3 | -22.3 |
| Plant height (cm) | -1.0 | 0.5 | 0.5 | -1.0 |
| Spike length (cm) | 2.0 | 6.1 | 2.0 | 1.0 |
| Spikelets per spike | -2.4 | 6.7 | -6.1 | -3.0 |
| Grains per spike | 0.8 | 1.8 | -6.2 | -3.5 |
| Biological yield (t ha^-1^) | 27.0 | 6.6 | 10.7 | -18.0 |
| Grain yield (t ha^-1^) | 27.3 | -3.0 | 24.2 | -21.2 |
| Straw yield (t ha^-1^) | 26.0 | 9.3 | 10.2 | -17.9 |
| 1000 grains weight (g) | 2.6 | 4.3 | 4.5 | 1.9 |
| Straw N content (%) | 107.7 | 97.4 | 56.4 | 38.5 |
| Grain N content (%) | 51.3 | 46.8 | 46.8 | 22.4 |
| N uptake straw (kg ha^-1^) | 159.6 | 114.2 | 71.1 | 13.5 |
| N uptake grain (kg ha^-1^) | 96.2 | 46.0 | 63.6 | -2.5 |
